# Triglycerides and blood pressure in relation to circulating CD34-positive cell levels among community-dwelling elderly Japanese men: a cross-sectional study

**DOI:** 10.1186/s12199-017-0684-x

**Published:** 2017-11-21

**Authors:** Yuji Shimizu, Shimpei Sato, Yuko Noguchi, Jun Koyamatsu, Hirotomo Yamanashi, Mako Nagayoshi, Koichiro Kadota, Shin-Ya Kawashiri, Yasuhiro Nagata, Takahiro Maeda

**Affiliations:** 1 Department of Community Medicine, Nagasaki University Graduate School of Biomedical Sciences, Nagasaki-shi, Sakamoto 1-12-4, Nagasaki, 852-8523 Japan; 2 Department of Cardiovascular Disease Prevention, Osaka Center for Cancer and Cardiovascular Disease Prevention, Osaka, Japan; 3 Department of Island and Community Medicine, Nagasaki University Graduate School of Biomedical Sciences, Nagasaki, Japan; 4 Center for Comprehensive Community Care Education, Nagasaki University Graduate School of Biomedical Sciences, Nagasaki, Japan

**Keywords:** CD34-positive cell, Hypertension, Triglycerides

## Abstract

**Background:**

Triglycerides are reported to be positively associated with blood pressure (both systolic and diastolic). However, in a previous study, we reported a significant positive association between triglycerides and circulating CD34-positive cells (endothelial repair) among non-hypertensive, but not hypertensive, participants. Since hypertension and endothelial dysfunction have a bi-directional association (vicious cycle), the status of circulating CD34-positive cells may influence the association between triglycerides and hypertension.

**Methods:**

Since antihypertensive medication use may influence results of the present study, we conducted a cross-sectional study of 327 community dwelling elderly (aged 60–69 years) Japanese participants who were not taking anti-hypertensive medication and who had participated in a general health check-up in 2013–2015.

**Results:**

Participants were classified into two groups based on median values of circulating CD34-positive cells (0.93 cells/μL). For participants with lower circulating CD34-positive cells (*n* = 165), a significant positive association was seen between triglycerides and blood pressure, but not for participants with higher circulating CD34-positive cells (*n* = 162). The multivariable standardized parameter estimates (β) and *p* values of systolic blood pressure and diastolic blood pressure were 0.23 (*p* = 0.007) and 0.18 (*p* = 0.036) for participants with lower circulating CD34-positive cells and 0.08 (*p* = 0.409) and 0.03 (*p* = 0.786) for those with higher circulating CD34-positive cells.

**Conclusion:**

A significant positive association between triglycerides and blood pressure exists among those with lower, but not higher, circulating CD34-positive cells. The level of circulating CD34-positive cells acts as a determinant factor for the association between triglycerides and blood pressure.

## Background

Triglycerides have been reported to be positively associated with systolic blood pressure and diastolic blood pressure in a community-dwelling sample of Japanese adults [1]. Since hypertension and endothelial dysfunction have a bi-directional association (vicious cycle) [2], increased endothelial repair activity may have a beneficial influence on preventing hypertension.

Participants with higher numbers of circulating CD34-positive cells may exhibit greater endothelial repair activity than those with lower circulating CD34-positive cells since such cells play an important role in endothelial repair [3, 4]. Our previous study showing a significant positive association between triglycerides and circulating CD34-positive cells (endothelial repair) among non-hypertensive, but not hypertensive, participants [5] partly supports a mechanism where higher endothelial repair activity may have a beneficial influence on preventing hypertension.

On the other hand, high-density lipoprotein (HDL) cholesterol from participants with cardiovascular disease is reported to be lacking in endothelial anti-inflammatory effects and does not stimulate endothelial repair [6]. In addition, high-density HDL cholesterol is positively associated with hypertension in participants with higher level of circulating CD34-positive cells [7] on the assumption that they might have a higher risk of cardiovascular disease which stimulates endothelial repair activity.

However, no studies have reported on the impact of circulating CD34-positive cell levels on the association between triglycerides and blood pressure.

Based on this mechanism, we hypothesized that triglycerides would be positively associated with blood pressure, but only in those with low levels of circulating CD34-positive cells, since they should have lower endothelial repair activity.

To clarify the association between triglycerides and blood pressure (systolic and diastolic), we conducted a cross-sectional study of 327 elderly men (aged 60–69 years) who were not taking anti-hypertensive medication and who had participated in an annual health check-up from 2013 to 2015.

## Methods

### Study population

The total number of male residents aged 60–69 in 2015 (estimated by the National Institute of Population and Social Security Research in March 2013) was 3264 for Goto city and 1010 for Saza town [8]. The study population comprised 617 male residents aged 60–69 years from Goto city and Saza town in the western part of Japan, who underwent an annual medical check-up from 2013 to 2015 as recommended by the Japanese government.

To avoid the influence of anti-hypertensive medication, participants on anti-hypertensive medication (*n* = 266) were excluded. Additionally, to avoid the influence of inflammatory disease and chronic disease (influence of undernutrition), participants with high white blood cell counts (≥ 10,000 cells/μL (*n* = 7)) and low body mass index (BMI < 18.5 kg/m^2^) (*n* = 14) were excluded from the analysis, as were persons with missing data (*n* = 3). The remaining patients, comprising 327 men at a mean age of 64.9 years (standard deviation (SD) 2.7; range 60–69), were enrolled in the study.

### Data collection and laboratory measurements

Trained interviewers obtained information on medical history. Current drinker (≥ 69 g/week) and current smoker were defined as drinker and smoker.

Body weight and height of patients wearing light clothing were measured using an automatic body composition analyzer (BF-220; Tanita, Tokyo, Japan), and BMI (kg/m^2^) was calculated. Fasting blood samples were collected in a heparin sodium tube, an EDTA-2K tube, and a siliconized tube. Fresh samples (within 24 h from drawing) from the heparin sodium tube were used to determine the number of CD34-positive cells. BD (Beckton Dickinson Biosciences) Trucount™ technology, an accurate and reproducible single platform assay cited in the International Society of Hematotherapy and Graft Engineering (ISHAGE) guidelines [9] and supported by automated software on the BD FACSCant™ II system, was used to measure circulating CD34-positive cells.

Samples from the EDTA-2K tube were used to measure white blood cell count using an automated procedure at SRL, Inc. (Tokyo, Japan). Serum triglycerides, serum HDL-cholesterol, hemoglobin A1c (HbA_1c_), and serum creatinine were measured using standard laboratory procedures at SRL, Inc. (Tokyo, Japan). Glomerular filtration rate (GFR) was estimated by using an established method recently proposed by a working group of the Japanese Chronic Kidney Disease Initiative [10]. According to this adaptation, GFR (ml/min/1.73 m^2^) = 194 × (serum creatinine (enzyme method))^−1.094^ × (age)^−0.287^.

### Statistical analysis

The median value for circulating CD34-positive cells (0.93 cells/μL) was set as the cutoff point, and we dichotomized the study population by median value.

Characteristics of the study population stratified by circulating CD34-positive cell levels were expressed as mean ± standard deviation. Simple correlation analysis and simple linear regression analysis for blood pressure (systolic and diastolic) and other variables were calculated for total participants and stratified by circulating CD34-positive cell levels. Multiple linear regression analyses were also performed to evaluate the same. Because triglycerides had a skewed distribution based on evaluation by the Kolmogorov-Smirnov test, logarithmic transformation was performed for the simple correlation analysis and linear regression analysis. All statistical analyses were performed with the SAS system for Windows (version 9.4; SAS Inc., Cary, NC). Probability values of less than 0.05 were considered to be statistically significant.

## Results

Among the whole study population, 165 participants were categorized as having lower CD34-positive cells (≤ 0.93 cells/μL) and 162 as higher CD34-positive cells (> 0.93 cells/μL).

Characteristics of the study population based on circulating CD34-positive cell levels are shown in Table 1. Compared to participants with low CD34-positive cell levels, those with high CD34-positive cell levels showed significantly higher BMI, prevalence of overweight, and triglyceride values, and significantly lower HDL cholesterol values. Since high BMI values are known to be associated with hypertension [11], we conducted further analyses and found that overweight participants (BMI ≥ 25 kg/m^2^) showed significantly higher triglycerides (129 ± 61 mg/dL) and lower HDL cholesterol (51.8 ± 9.4 mg/dL) compared to non-overweight participants (108 ± 81 mg/dL), *p* = 0.037, and (59.0 ± 15.0 mg/dL), *p* < 0.001, respectively.

**Table 1:** Characteristics of the study population based on levels of circulating CD34-positive cells

	CD34-positive cells		*p*
	Lower	Higher	
Circulating CD34 positive cells, cells/μL	0.63 ± 0.19	2.02 ± 1.82	
No. of participants	165	162	
Age, year	65.0 ± 2.6	64.9 ± 2.7	0.600
Systolic blood pressure, mmHg	131 ± 18	133 ± 18	0.312
Diastolic blood pressure, mmHg	79 ± 12	80 ± 12	0.403
BMI, kg/m^2^	22.6 ± 2.6	23.7 ± 2.7	< 0.001
Over weight (BMI ≥ 25 kg/m^2^), %	17.6	30.9	0.005
Drinker, %	43.6	45.1	0.796
Smoker, %	12.7	17.9	0.195
HDL, mg/dL	59 ± 13	55 ± 15	0.019
Triglycerides, mg/dL	95 ± 46	131 ± 96	< 0.001
HbA_1c_, %	5.6 ± 0.6	5.7 ± 0.7	0.159
Serum creatinine, mg/dL	0.85 ± 0.16	0.83 ± 0.14	0.183
GFR, mL/min/1.73m^2^	72.0 ± 13.3	73.8 ± 13.1	0.222

Values, mean ± standard deviation. Lower CD34-positive cells, ≤ 0.93 cells/μL. Higher CD34-positive cells, > 0.93 cells/μL

*BMI* body mass index, *HDL* HDL cholesterol, *HbA*_*1c*_ hemoglobin A_1c_, *GFR* glomerular filtration rate

Table 2 shows a simple correlation analysis of blood pressure and other variables.

**Table 2:** Simple correlation coefficient of blood pressure (systolic and diastolic) and other variables

	Systolic blood pressure			Diastolic blood pressure		
		CD34-positive cells			CD34-positive cells	
	Total participants	Lower	Higher	Total participants	Lower	Higher
No. of participants	327	165	162	327	165	162
Age	0.03	− 0.01	0.07	− 0.07	− 0.08	− 0.06
	(0.586)	(0.901)	(0.357)	(0.187)	(0.278)	(0.455)
BMI	0.17	0.20	0.13	0.22	0.25	0.18
	(0.002)	(0.009)	(0.099)	(< 0.001)	(0.001)	(0.022)
Drinker	0.14	0.14	0.14	0.04	0.02	0.07
	(0.010)	(0.073)	(0.066)	(0.441)	(0.821)	(0.391)
Smoker	− 0.05	− 0.05	− 0.06	0.02	0.16	− 0.11
	(0.344)	(0.523)	(0.425)	(0.721)	(0.046)	(0.156)
HDL	0.05	− 0.02	0.12	0.04	− 0.03	0.12
	(0.413)	(0.837)	(0.142)	(0.438)	(0.729)	(0.126)
HbA_1c_	0.13	0.23	0.03	0.08	0.10	0.05
	(0.023)	(0.004)	(0.691)	(0.156)	(0.181)	(0.532)
GFR	− 0.006	− 0.04	0.02	− 0.03	− 0.01	− 0.05
	(0.910)	(0.629)	(0.809)	(0.610)	(0.897)	(0.490)
Triglycerides	0.13	0.26	0.003	0.08	0.17	− 0.02
	(0.019)	(< 0.001)	(0.962)	(0.164)	(0.026)	(0.772)

Triglycerides are calculated in logarithm values. Lower CD34-positive cells, ≤ 0.93 cells/μL. Higher CD34-positive cells, > 0.93 cells/μL. The *p* values are enclosed in parentheses

*BMI* body mass index, *HDL* high-density lipoprotein cholesterol, *HbA*_*1c*_ hemoglobin A_1c_, *GFR* glomerular filtration rate

For participants with lower CD34-positive cell levels, a significant positive association was seen between triglycerides and systolic and diastolic blood pressure, whereas no significant associations were observed for participants with higher levels of CD34-positive cells. The simple correlation coefficients and *p* values of systolic blood pressure and diastolic blood pressure for participants with lower CD34-positive cell levels were 0.26 (*p* = 0.001) and 0.17 (*p* = 0.026), respectively, and for participants with higher CD34-positive cell levels were 0.003 (*p* = 0.962) and − 0.02 (*p* = 0.772), respectively.

**Fig. 1:**
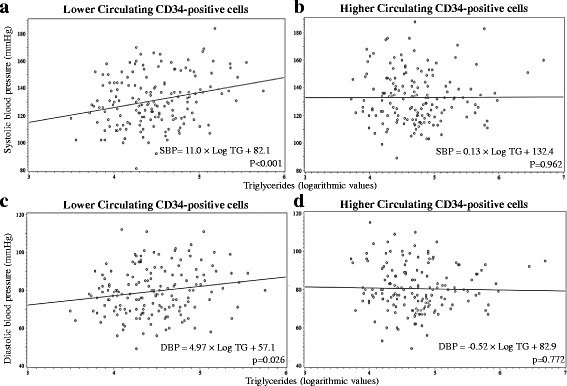
Scatter plot of blood pressure (systolic and diastolic) and triglycerides

Figure 1 shows the circulating CD34-positive cell level-specific correlation between blood pressure and triglycerides from a simple linear regression analysis. For participants with lower circulating CD34-positive cells, a significant positive association was seen between triglycerides and systolic and diastolic blood pressure, whereas no significant associations were observed for participants with higher circulating CD34-positive cells. These correlations remained unchanged even after further adjustment for known cardiovascular risk factors (Table 3). The multivariable standardized parameter estimates and *p* values of triglycerides for systolic and diastolic blood pressure were 0.23 (*p* = 0.007) and 0.18 (*p* = 0.036), respectively, for lower circulating CD34-positive cells and 0.08 (*p* = 0.409) and 0.03 (*p* = 0.786), respectively, for higher circulating CD34-positive cells.

**Table 3:** Multiple linear regression analysis of blood pressure (systolic and diastolic) with relevant factors adjusted for confounding factors

	Total participants			CD34-positive cells					
				Lower			Higher		
	*Β*	*β*	*p*	*Β*	*β*	*p*	*Β*	*β*	*p*
Systolic blood pressure									
No. of participants	327			165			162		
Age	0.37	0.05	0.333	− 0.03	− 0.004	0.960	0.72	0.11	0.180
BMI	1.13	0.17	0.004	1.06	0.15	0.061	1.23	0.18	0.031
Drinker	3.93	0.11	0.070	3.35	0.09	0.274	3.44	0.09	0.273
Smoker	− 0.02	− 0.0005	0.993	1.15	0.02	0.793	− 1.05	− 0.02	0.790
HDL	0.21	0.16	0.013	0.15	0.11	0.196	0.25	0.20	0.036
HbA_1c_	2.78	0.10	0.066	6.01	0.20	0.009	0.49	0.02	0.809
GFR	− 0.02	− 0.02	0.765	0.02	0.01	0.875	− 0.02	− 0.02	0.831
Triglycerides	4.80	0.13	0.035	9.61	0.23	0.007	2.67	0.08	0.409
Diastolic blood pressure									
No. of participants	327			165			162		
Age	− 0.22	− 0.05	0.383	− 0.28	− 0.06	0.462	− 0.08	− 0.02	0.809
BMI	1.02	0.22	< 0.001	1.10	0.23	0.005	1.04	0.24	0.005
Drinker	0.39	0.02	0.790	0.015	0.001	0.994	0.25	0.01	0.903
Smoker	1.04	0.03	0.595	6.62	0.18	0.028	− 2.93	− 0.09	0.257
HDL	0.13	0.15	0.022	0.08	0.08	0.326	0.16	0.20	0.037
HbA_1c_	1.03	0.06	0.307	1.66	0.08	0.282	0.69	0.04	0.606
GFR	− 0.03	− 0.03	0.535	0.04	0.04	0.625	− 0.07	− 0.08	0.333
Triglycerides	1.85	0.08	0.225	5.04	0.18	0.036	0.57	0.03	0.786

Triglycerides are calculated in logarithm values. Lower CD34-positive cells, ≤ 0.93 cells/μL. Higher CD34-positive cells, > 0.93 cells/μL

*BMI* body mass index, *HDL* high-density lipoprotein cholesterol, *HbA*_*1c*_ hemoglobin A1c, *GFR* glomerular filtration rate, *Β* parameter estimate, *β* standardized parameter estimate, *p p* factor for multivariable linear regression models

Since our previous study that reported an association between triglycerides and circulating CD34-postive cells did not use the log transformation technique for triglycerides [2], we performed further analyses without using log transformation and found essentially the same associations. The simple correlation coefficient (*r*) for systolic blood pressure and triglycerides was *r* = 0.27 (*p* = 0.005) for participants with lower CD34-positive cells and *r* = 0.07 (*p* = 0.351) for subjects with higher CD34-positive cells; the simple correlation coefficient (*r*) for diastolic blood pressure and triglycerides was *r* = 0.16 (*p* = 0.040) for participants with lower CD34-positive cells and *r* = 0.06 (*p* = 0.486) for participants with higher CD34-positive cells. From a multivariable linear regression model, the parameter estimate (*Β*) and standardized parameter estimate (*β*) for systolic blood pressure was *Β* = 0.09 and *β* = 0.24 (*p* = 0.004) for participants with lower CD34-positive cells and *Β* = 0.03 and *β* = 0.16 (*p* = 0.072) for participants with higher CD34-positive cells; the parameter estimate (*Β*) and standardized parameter estimate (*β*) for diastolic blood pressure was *Β* = 0.04 and *β* = 0.16 (*p* = 0.049) for participants with lower CD34-positive cells and *Β* = 0.01 and *β* = 0.12 (*p* = 0.182) for participants with lower CD34-positive cells.

We also uncovered a significant association between triglycerides and CD34-positive cell level on systolic blood pressure and diastolic blood pressure, with *p* values for the effect of this interaction of 0.011 and 0.049, respectively.

## Discussion

The major findings of present study are that for participants with lower circulating CD34-positive cells, independent of known cardiovascular risk factors, triglycerides are positively associated with blood pressure, but not in participants with higher circulating CD34-positive cells. The level of circulating CD34-positive cells acts as a determinant factor for the association between triglycerides and blood pressure.

A previous Japanese study of 567 men and 808 women aged 19–90 years reported an independent positive association between triglycerides and systolic blood pressure and diastolic blood pressure [1]. That study is partly compatible with our results showing, independent of known cardiovascular risk factors, a significant positive association between triglycerides and systolic blood pressure, but not diastolic blood pressure, in total participants.

We found further evidence that circulating CD34-positive cell levels may influence the association between triglycerides and blood pressure, since triglycerides were found to be positively associated with systolic and diastolic blood pressure in participants with lower, but not higher, circulating CD34-positive cells.

Hypertension and endothelial dysfunction have a bi-directional association (vicious cycle) [2]. Since triglycerides can become an independent risk factor for the early development of atherosclerosis (endothelial dysfunction) [12, 13], triglycerides may be positively associated with blood pressure.

However, such positive associations were not observed among participants with higher circulating CD34-positive cells. Bone marrow-derived endothelial progenitor cells such as CD34-positive cells have been reported to play an important role in maintaining the vascular endothelium [14, 15], and endothelial dysfunction has been recognized as one of the initial mechanisms leading to atherosclerosis (increased arterial stiffness) [16]. Therefore, increased levels of circulating CD34-positive cells should have a beneficial effect in preventing atherosclerosis (endothelial dysfunction) in those where no significant association exists between triglycerides and hypertension. A previous study of 57 asymptomatic men that reported an association between increased numbers of circulating CD34-positive cells and a decrease in the extent of subclinical atherosclerosis [17] might support the above-mentioned mechanisms.

Although increased levels of circulating CD34-positive cells should have a beneficial effect on the endothelium, our present study showed essentially the same values for blood pressure regardless of CD34-positive cell level. Our previous study reported a positive association between BMI and circulating CD34-positive cell levels, divided by median values of circulating CD34-positive cells among the general population [2], as in the present study. Since BMI is positively associated with hypertension [11], and hypertension participates in a vicious cycle with endothelial dysfunction [2], overweight participants (BMI ≥ 25 kg/m^2^) should have activated endothelial activity, resulting in elevated circulating CD34-positive cells. In our present study, overweight participants showed significantly higher values for triglycerides and lower values for HDL cholesterol compared to non-overweight participants. Therefore, participants with higher CD34-positive cells could have significantly higher triglycerides and lower HDL cholesterol due to a higher prevalence of being overweight. In fact, the present study found that participants with higher CD34-positive cells show a significantly higher prevalence of being overweight compared to those with lower CD34-positive cells. Since higher BMI is associated with hypertension [11], and HDL cholesterol is reported to be positively associated with hypertension in participants with higher, but not lower, circulating CD34-positive cells [7], these factors might cause elevated blood pressure values in participants with higher circulating CD34-positive cells. In addition to the influence of a higher prevalence of being overweight, unlike HDL cholesterol from healthy participants, HDL cholesterol from participants with stable cardiovascular disease or acute coronary syndrome is reported to be lacking in endothelial anti-inflammatory effects and does not stimulate endothelial repair [6]. Therefore, participants with a high level of CD34-positive cells should present with endothelial injury similar to participants with stable cardiovascular disease and acute coronary syndrome since endothelial repair activity should be stimulated by endothelial injury.

Potential limitations of this study warrant consideration. Although the level of circulating CD34-positive cells significantly influences the association between triglycerides and blood pressure, no data on the evaluation of endothelial function was available. Further analyses that include endothelial function-related data such as flow-mediated dilation (FMD) will be necessary. Additionally, because this was a cross-sectional study, causal relationships were not able to be established.

## Conclusion

In conclusion, for participants with lower circulating CD34-positive cells, independent of known cardiovascular risk factors, triglycerides are positively associated with blood pressure, but not in participants with higher circulating CD34-positive cells. The level of circulating CD34-positive cells acts as a determinant factor for the association between triglycerides and blood pressure.

## Abbreviations

BMI: Body mass index
FMD: Flow-mediated dilation
GFR: Glomerular filtration rate
HbA_1c_: Hemoglobin A_1c_
HDL: High-density lipoprotein cholesterol
SD: Standard deviation
r: Correlation coefficient
Β: Parameter estimate
β: Standardized parameter estimate
